# Recent Advances in Metal Phthalocyanine for Sensing Applications

**DOI:** 10.3390/nano16050312

**Published:** 2026-02-28

**Authors:** Hao Wu, Qifubo Geng, Xunjun He, Mingze Zhang, Sergey Maksimenko

**Affiliations:** 1 School of Electrical and Electronic Engineering, Harbin University of Science and Technology, Harbin 150080, China; 2 School of Information Engineering, Minzu University of China, Beijing 100081, China; 3 Research Institute for Nuclear Problems, Belarusian State University, 220006 Minsk, Belarus

**Keywords:** metal phthalocyanine, photoelectric sensor, gas sensor, biosensing sensor

## Abstract

In recent years, metal phthalocyanine (MPc)-based sensors have garnered significant interest for applications in environmental monitoring, biomedical diagnostics, and industrial process control, owing to their efficient and cost-effective sensing capabilities. In contrast to conventional inorganic materials, MPcs are a class of small-molecule materials characterized by a stable, π-conjugated macrocyclic framework with a tunable central metal ion. This structural architecture imparts unique physicochemical properties, including high chemical stability, excellent redox activity, structural versatility, considerable dielectric constant and electrical conductivity, along with pronounced optical absorption and excellent environmental stability. By incorporating different metal ions into the macrocyclic core, their functional characteristics can be precisely modulated to achieve high sensitivity and selectivity toward various gas, ion, or biomolecule targets. Leveraging these advantages, MPcs have been extensively utilized in diverse sensing platforms, such as photoelectric, gas, and biosensors. This review outlines recent advances in MPc-based sensor research and provides perspectives on their future development trends.

## 1. Introduction

**Figure 1:**
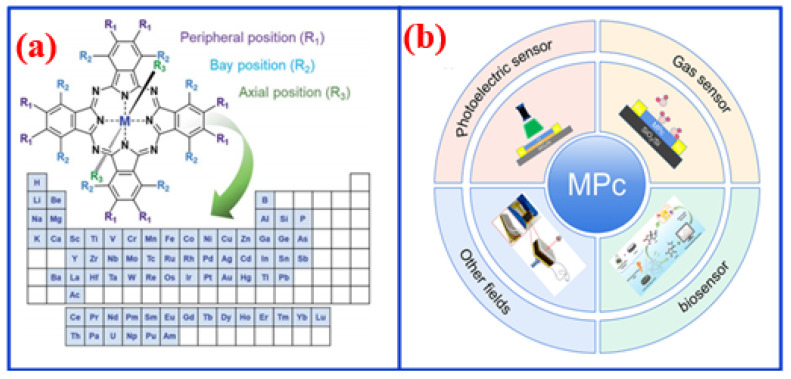
(**a**) Molecular structures [1] and (**b**) application fields of MPcs.

Metal phthalocyanines (MPcs) are a class of small-molecule materials composed of four isoindole units bridged by nitrogen atoms, forming an 18π-electron aromatic system. These macrocycles coordinate a central metal or metalloid ion via two covalent and two coordination bonds. Since their discovery in the late 19th century, MPcs have been extensively used in dyes, inks, plastics, catalysis, and photochromic materials due to their intense coloration. As organic small molecules, MPcs can form stable thin films through vacuum evaporation and are widely employed in organic optoelectronic devices. To tailor device performance, various metal centers, such as Cu, Fe, Zn, Sn, Mn, Co, Ni, and Pb, can be incorporated into the MPc framework, as illustrated in Figure 1a. Given the availability of nearly 70 metal ions and 16 possible substitution sites at peripheral and bay positions, a wide variety of MPc complexes can be synthesized, each exhibiting distinct properties depending on the selected metal center [1].

Compared with traditional inorganic materials, MPcs are regarded as an ideal class of sensing materials for several key reasons. First, their unique 18π-conjugated macrocyclic structure effectively lowers the energy barrier to charge-carrier transport. This contrasts with other porous or framework materials, such as MOFs, COFs, and imine compounds, which primarily enhance specific surface area without inherently facilitating charge migration [2,3]. The reduced energy barrier in MPcs promotes efficient carrier transport, thereby improving both the detection capability and response speed of MPc-based sensors [4,5]. Moreover, MPcs exhibit strong light absorption across the visible and near-infrared regions, rendering them well-suited for photoelectric sensing. For example, copper phthalocyanine (CuPc) displays distinct absorption bands in the 300–400 nm and 600–700 nm ranges, spanning a broader spectrum than conventional semiconductors like GaN and TiO_2_ [6,7,8,9].

In addition to these electronic and optical advantages, MPcs offer practical benefits in terms of processing and cost. They can be synthesized at relatively low temperatures from readily available precursors and are compatible with low-cost fabrication techniques, including thermal evaporation and liquid-phase exfoliation. These features render MPc-based sensors more economical to produce than many alternatives [10,11,12,13,14]. Moreover, MPcs function as excellent p-type semiconductors and are widely used as hole-transport layers in solar cells [15]. They have also been successfully implemented as active sensing materials in various devices, including gas sensors, cannabis sensors, and electrochemical sensors [16,17,18]. Beyond sensing applications, MPcs demonstrate notable catalytic activity and have been applied in a range of photocatalytic reactions [19,20,21,22].

This review focuses on the sensing performance of MPcs and summarizes recent advances in their application across various sensor platforms, including photoelectric, gas, and biosensors, as well as in related fields like piezoelectrics. Key device architectures and core performance metrics are discussed, current challenges are identified along with practical recommendations, and forward-looking perspectives on the future development of MPc-based sensing systems are provided.

## 2. Sensing Mechanism and Application of MPcs

**Figure 2:**
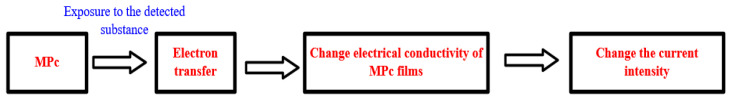
Sensing mechanism of MPcs.

The versatile sensing capabilities of MPcs originate from their distinctive molecular and electronic structure. The extended 18π-electron aromatic macrocycle interacts strongly with various analytes, inducing measurable changes in electrical conductivity, optical absorption, or mass. These interactions, which can involve charge transfer, chemical binding, or physical adsorption at the central metal ion or the organic ligand, effectively modulate the electrical or optical properties of MPcs. For example, upon exposure to a target substance, electron transfer between the analyte and the π-electron system or the central metal ion alters the electrical conductivity of the materials, thereby modulating the current and enabling sensing functionality [23,24,25], as illustrated in Figure 2. In addition to their responsive nature, MPcs exhibit excellent thermal and chemical stability, ensuring reliable performance across diverse environments. This adaptable sensing mechanism, combined with molecular tailorability, allows MPcs to be designed for the detection of a broad spectrum of analytes, rendering them highly attractive for practical sensing applications. As shown in Figure 1b, they are effectively employed in gas sensors for pollutants like NO_2_, NH_3_, and volatile organic compounds; in photodetectors and optical sensors, leveraging their strong and tunable light absorption; in biosensors for glucose or cholesterol monitoring via electrochemical activity; and in piezoelectric sensors where their thin-film morphology can transduce mechanical signals.

## 3. Photoelectric Sensors

Photoelectric sensors, also known as photodetectors, are photoelectric devices that detect the presence, absence, or distance to a target object by using a light source and a photoelectric receiver. They operate by converting incident light into corresponding electrical outputs. Recognized for their high speed, non-contact operation, long detection range, and precision, these sensors are widely used in industrial automation, robotics, security systems, and consumer electronics. Based on operating wavelength, photodetectors are generally categorized into near-infrared (NIR), visible (Vis), ultraviolet (UV), and broadband ultraviolet–visible–near-infrared (UV–Vis–NIR) photodetectors.

### 3.1. NIR Photodetectors

**Figure 3:**
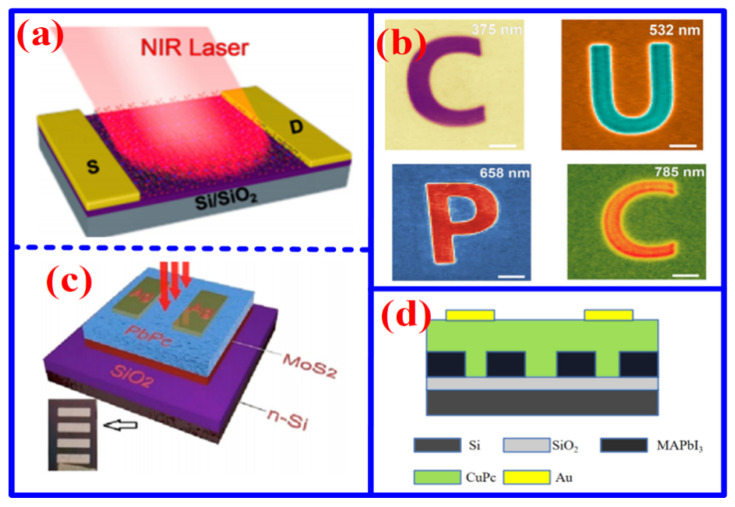
(**a**) Schematic diagram and (**b**) various wavelength-excited photocurrent imaging of CuPc NIR sensor [29]. (**c**) Schematic diagram of PbPc/MoS_2_ NIR photodetector [31]. (**d**) Schematic diagram.

The NIR spectral region (760–2500 nm), distinguished by its unique optical characteristics, is widely regarded as a highly promising candidate for imaging applications. In contrast to visible light, NIR radiation offers greater penetration depth and lower attenuation in biological tissues due to diminished absorption and scattering. These characteristics enable NIR-based technologies to achieve high signal-to-noise ratios and deep-tissue imaging, positioning NIR as a compelling platform for next-generation non-invasive health monitoring and high-resolution functional imaging. To date, a variety of material strategies have been explored for developing high-performance NIR sensors [26,27,28]. For instance, Lu et al. reported an NIR polarization sensor based on a CuPc film, which demonstrated a responsivity of 200 A/W and clear imaging capability (Figure 3a,b) [29]. Nevertheless, single-material sensors often encounter inherent limitations in carrier transport and spectral absorption. To address this challenge, Xu et al. developed a PbPc/MoS_2_ heterojunction NIR photodetector, achieving a peak responsivity of 1263.8 A/W under 808 nm illumination (Figure 3c) [30,31]. The enhanced responsivity is attributed to the heterojunction architecture, which effectively broadens the spectral response, facilitates exciton separation, and suppresses carrier recombination.

To further address the limitations of limited specific surface area and poor strain tolerance in conventional thin films, Lu et al. employed a DVD-based method to fabricate a CuPc/MAPbI_3_ nanoarray NIR sensor (Figure 3d) [32]. In contrast to traditional film structures, the nanowire architecture not only offers a larger specific surface area, improving interaction with incident light, but also shortens the carrier transport path, thereby enabling more efficient carrier movement. The device achieved a responsivity of 84.5 A/W, a detectivity of 1.94 × 10^13^ Jones, and an external quantum efficiency of 1.97 × 10^4^% at 532 nm, demonstrating outstanding photoelectric performance across both NIR and visible regions. Furthermore, this DVD-assisted approach provides a reproducible route to the synthesis of self-oriented nanostructures. Despite notable progress in MPc-based NIR detection, the inherently low electrical conductivity of these materials still constrains the sensitivity of NIR sensors. Accordingly, addressing this limitation through doping strategies to enhance charge transport is a critical direction for future research.

### 3.2. Vis Photodetectors

With recent advances in solar cells and imaging technologies, MPc-based Vis photodetectors have attracted considerable attention. For example, Vijayan et al. reported a CuPc-based photodetector exhibiting a responsivity of 1.14 A/W and a specific detectivity of 0.02 × 10^11^ Jones under Vis-light illumination [33]. Similarly, Zhang et al. developed a ZnPc-based Vis photodetector achieving a responsivity of 5.35 A/W [34]. To further improve device performance, transition metal dichalcogenides (TMDs) have been integrated with MPcs to form heterojunctions. Owing to the excellent photoelectric properties of MoS_2_, the majority of reported MPc-based Vis photodetectors employ a CuPc/MoS_2_ thin-film architecture [35,36,37,38,39]. For instance, Pak et al. fabricated a CuPc/MoS_2_ heterojunction photodetector that achieved a responsivity of 1.98 A/W and a detectivity of 1.49 × 10^10^ Jones under 405 nm illumination (40 mW), and further elucidated the underlying operating mechanism of the device [40,41].

To further optimize device architecture, Xu et al. fabricated a high-performance CuPc/MoS_2_ Vis photodetector using a CVD-grown single-layer MoS_2_ film [42]. The device exhibited remarkable performance metrics, including a detectivity of 2 × 10^10^ Jones, a responsivity of 3 × 10^3^ A/W, a fast response time of 436 µs, and an external quantum efficiency of up to 483% across the visible spectrum. Analysis revealed that the built-in electric field at the heterojunction interface promotes efficient exciton dissociation, significantly enhancing both detectivity and responsivity. Moreover, the high carrier mobility of the single-layer MoS_2_ contributed to the markedly reduced response time. These advantages render the device promising for applications in high-speed data transmission and non-destructive monitoring systems. However, the limited mechanical robustness of thin-film structures restricts their applications in flexible sensing. To overcome this limitation, research has increasingly shifted toward nanowire-based architectures [43,44,45,46]. For instance, Liao et al. developed a Vis photodetector based on self-oriented CuPc nanowires [47]. By integrating these nanowires into a 10 × 10 electrode array, the photodetector achieved faster response speeds (rise time of 0.05–0.43 s and fall time of 0.38–2.34 s) across the 488–780 nm range, outperforming conventional CuPc devices fabricated via other methods. The device also achieved an average detectivity of 2.49 × 10^10^ Jones. These improvements are attributed to the nanowire geometry, which provides a larger interface for light-matter interaction and shortens carrier migration pathways.

**Figure 4:**
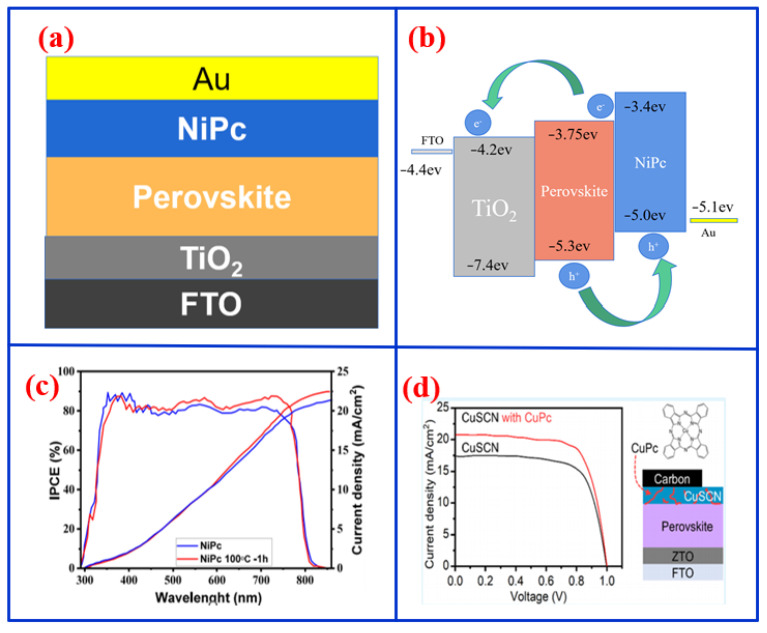
(**a**) Structure, (**b**) energy band and charge transfer, and (**c**) IPCE and current density of perovskite solar cells with NiPc acting as the HTM [50]. (**d**) Current density and structure of the solar cell with and without CuPc [55].

In addition to their conventional applications in Vis photodetectors, MPcs have also been effectively utilized as hole-transport layers (HTLs) to enhance the performance and stability of solar cells [48]. For example, Rawat et al. incorporated a NiPc film as a hole interface buffer layer in P3HT:PCBM solar cells, achieving a 65% increase in power conversion efficiency (PCE), reaching 3.17% [49]. The performance improvement can be attributed to the optimized NiPc layer inserted between PEDOT:PSS and the active layer, which reduces undesirable charge recombination losses at the interfaces and results in the lower series and shunt resistances. Haider et al. employed NiPc as an HTL in perovskite solar cells to improve the NiPc/perovskite interface and promote charge transport (Figure 4a) [50]. The band structure illustrated in Figure 4b indicates that NiPc facilitates carrier transport between the valence and conduction bands. Consequently, the IPCE of solar cells with NiPc achieved approximately 80%, and the current intensity after annealing at 100 °C for one hour remained 23 mA/cm^2^ (Figure 4c). Zou et al. fabricated a ZnPc-based solar cell integrated with CsPbBr_3_ quantum dots, achieving a PCE of 10.20% along with long-term stability [51]. Further improvements have been realized through molecular engineering of MPc-based HTLs. Xu et al. designed a novel phthalocyanine derivative (TQ) as an HTL, which increased device efficiency from 21.91% to 24.29% by enhancing hole mobility, conductivity, and the built-in electric field [52]. Qu et al. developed a methylthiotrianiline-substituted copper phthalocyanine HTL, achieving a PCE of 23.0% and exceptional thermal stability (retaining 96% of its initial efficiency after 3624 h at 85 °C) owing to effective defect passivation [53]. Collectively, these studies demonstrate that MPc-based HTLs can effectively improve interfacial properties and charge management in photovoltaic devices.

To further address limitations in surface passivation and suppress charge recombination in single-layer HTLs, hybrid and composite HTLs incorporating MPcs have been developed to serve as functional interlayers. For instance, Murokinas et al. employed a vapor-deposited CuPc/MS-OC bilayer as the HTL in solar cells, achieving a PCE of 14.42% while maintaining stable performance over 400 h of continuous illumination [54]. Makming et al. integrated CuPc with copper (I) thiocyanate (CuSCN) to form a hybrid HTL, attaining a PCE of 15.01% under AM1.5G illumination and 32.1% under 1000 lux, along with the current density exceeding 20 mA/cm^2^ (Figure 4d) [55]. Similarly, Zhao et al. designed a photo-conductive NiOx-Pc composite HTL to overcome the intrinsically low conductivity of NiOx [56]. Under illumination, charge transfer from Pc to NiOx enhanced the hole density and conductivity by approximately 300%, yielding a PCE of 21.68% and high thermal stability (retaining over 95% of the initial efficiency after 800 h at 85 °C).

### 3.3. UV Photodetectors

**Figure 5:**
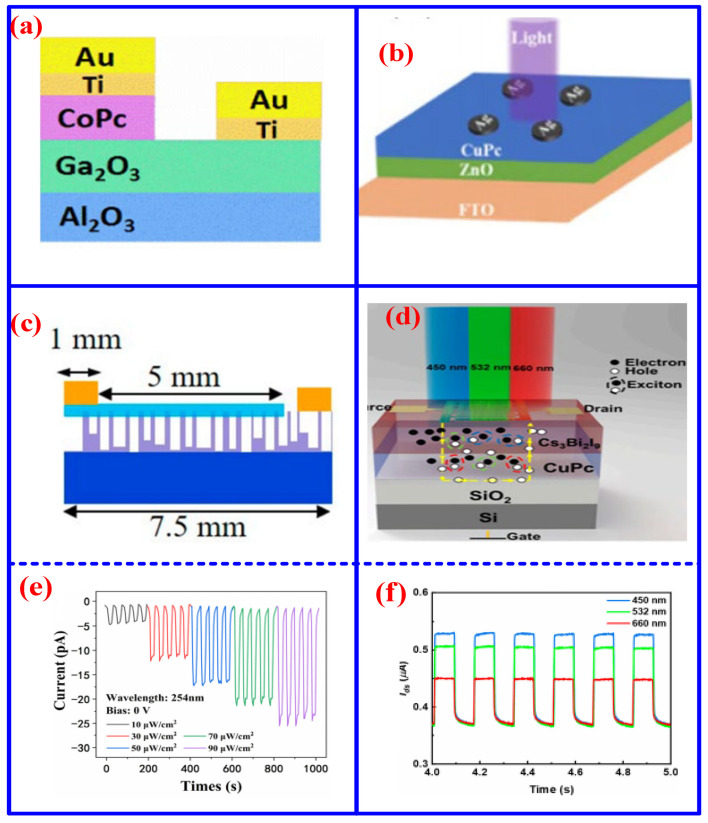
Schematic diagram of various structural UV photodetectors: (**a**) CoPc/CoO_3_ [65], (**b**) CuPc/ZnO [66], (**c**) CuPc/porous-GaN [68], and (**d**) CuPc/Cs_3_Bi_2_I_9_ (The existing excition within yellow dash line) [78], (**e**,**f**) The graph of current variation with time of CoPc/porous-GaN [68] and CuPc/Cs_3_Br_2_I_3_ [78] at different optical powers and different wavelengths.

Compared to their Vis photodetectors, UV photodetectors provide distinct advantages, largely owing to their selective spectral sensitivity. Their key strength lies in high sensitivity to UV radiation while effectively suppressing interference from the stronger visible and infrared components present in ambient light, thereby achieving a superior signal-to-noise ratio in scenarios where Vis detectors would be prone to saturation or ineffective detection. Specifically, UV photodetectors can operate within the solar-blind region (200–280 nm), where solar radiation is largely absorbed by the Earth’s atmosphere [57,58,59,60,61]. This enables high-sensitivity detection of UV sources, such as flames, electrical coronas, or optical signals, against a near-zero background, even under full daylight conditions. Moreover, typically fabricated from wide-bandgap semiconductors, these devices inherently exhibit low dark current and high thermal stability, contributing to enhanced sensitivity and operational robustness [62,63,64]. These characteristics render UV photodetectors indispensable for applications in which Vis detectors would be ineffective or become saturated, including environmental and safety monitoring, military and aerospace systems, and scientific and medical instrumentation. Recently, various MPc-based UV photodetectors have garnered considerable attention. For instance, Qi et al. developed a CoPc/Ga_2_O_3_ type-II heterojunction UV photodetector (Figure 5a), wherein the heterojunction effectively suppresses dark current and reduces noise, achieving an ultralow dark current of 5.7 fA, a responsivity of 18.4 mA/W, and a detectivity of 1.92 × 10^17^ Jones [65]. In another example, Chu et al. reported a self-powered UV photodetector based on ZnO and CuPc (Figure 5b) [66]. Under 365 nm illumination at a bias of 2 V, the device demonstrated a detectivity of 7.63 × 10^11^ Jones, a responsivity of 227.11 mA/W, and an external quantum efficiency (EQE) of 77.23%. Despite these advances, such UV sensors are often hindered by slow response speeds and limited responsivity.

To further enhance device performance, UV photodetectors incorporating porous structures have been successively designed and developed. For instance, Peng et al. reported a CuPc/porous GaN-based UV photodetector that achieved a high on/off ratio of 162 [67]. Similarly, Xiao et al. fabricated a self-powered CoPc/porous GaN photodetector, attaining an on/off ratio of 105, a responsivity of 588 mA/W, and a detectivity of 4.8 × 10^12^ Jones (Figure 5c) [68]. The porous architecture, analogous to a nanowire network, promotes exciton separation at the interface and shortens the carrier diffusion distance within the material, thereby significantly increasing the effective interfacial area and reducing the carrier transmission path. As illustrated in Figure 5e, this device exhibits a fast photoresponse under different optical power levels. Beyond porous designs, perovskites have emerged as a promising material platform for UV detection due to their outstanding photoelectric properties [69,70,71]. In 2023, Wang et al. developed a CuPc/Cs_3_Bi_2_I_9_-based phototransistor with a broad spectral response spanning the UV to Vis region, achieving a responsivity of 1.96 A/W and a detectivity of 6.91 × 10^11^ Jones at 450 nm (Figure 5d) [72]. Figure 5f shows the time-dependent photocurrent response of the CuPc/Cs_3_Br_2_I_3_ device under different wavelength illuminations. Moreover, the device exhibited remarkable environmental stability, retaining its performance over 360 h in ambient air. To further demonstrate the advantages of MPc-based UV photodetectors, a comparison of key performance metrics for UV photodetectors based on different materials is summarized in Table 1. The comparison results reveal that MPc-based devices generally exhibit lower responsivity than their inorganic counterparts, yet achieve higher detectivity. This performance trade-off can be attributed to the inherently higher carrier density of inorganic semiconductors, whereas organic materials often possess fewer defects, thereby contributing to reduced noise and enhanced detectivity [73,74,75].

### 3.4. UV–Vis–NIR Photodetectors

**Figure 6:**
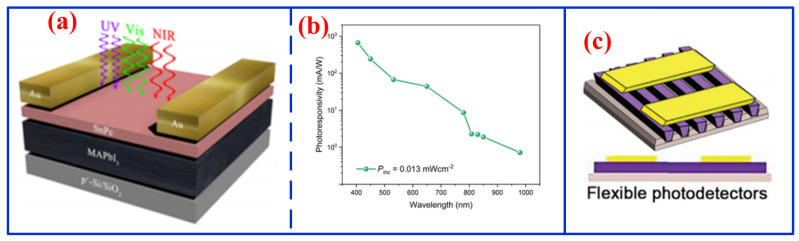
(**a**) Schematic diagram and (**b**) responsivity of SnPc/MAPbI_3_ UV–Vis–NIR photodetector [85] and (**c**) schematic diagram of flexible F_16_CuPc UV–Vis–NIR photodetector [86].

MPc-based photodetectors, as aforementioned, are typically constrained to narrow spectral ranges, often necessitating the use of multiple discrete devices for full-spectrum analysis, thereby increasing system complexity and cost. In contrast, photodetectors capable of operating across the UV–Vis–NIR spectrum offer a significantly broader detection band relative to single-band counterparts, enabling the acquisition of more comprehensive optical information. Moreover, these devices exhibit excellent environmental stability and are simultaneously applicable to water quality monitoring and atmospheric sensing [76]. In response to the inherent limitations of conventional single-band photodetectors, considerable research efforts have been increasingly directed toward the development of integrated systems capable of full-spectrum detection [77,78]. For instance, Huang et al. reported a SnPc/MAPbI_3_-based photodetector with response spanning the UV, Vis, and NIR regions (Figure 6a) [79]. The device achieved a detectivity of 6.45 × 10^12^ Jones and a responsivity of 665.75 mA/W, along with outstanding operational stability over 200 h, demonstrating its strong potential for full-spectrum imaging applications (Figure 6b). Similarly, Liu et al. fabricated a flexible photodetector based on F_16_CuPc on a PDMS substrate (Figure 6c) [80], achieving a responsivity of 1.1 × 10^−4^ A/W and a detectivity of 2.6 × 10^9^ Jones across the UV–Vis–NIR range. Moreover, this fabrication strategy has been successfully extended to other MPcs, including ZnPc, FePc, CoPc, and NiPc. At present, MPc-based UV–Vis–NIR photodetectors have evolved from conventional thin-film architectures to self-oriented nanowire structures, indicating a clear trend toward wearable and flexible device applications. These advances highlight their considerable promise for emerging applications in human health monitoring and environmental protection.

Despite significant progress, MPc-based photodetectors still confront critical limitations, including low charge-carrier mobility, inefficient exciton dissociation, inadequate environmental stability, and narrow spectral response. To address these limitations, a range of strategies has been actively pursued, encompassing molecular engineering of MPc structures, formation of heterojunctions, optimization of device architecture, and advanced encapsulation. These approaches synergistically enhance responsivity, response speed, and operational lifetime. Future studies will focus on the design of novel MPc derivatives with enhanced stability, the development of hybrid systems incorporating perovskites or plasmonic nanostructures, and the integration of these detectors into flexible and wearable electronic platforms. Concurrent efforts are focused on the realization of monolithic systems that combine photodetection with on-chip signal processing. Furthermore, exploration of alternative operating mechanisms, such as photomultiplication effects in MPc-based devices, could unlock further functionalities for specialized and cost-effective optoelectronic applications. In summary, while challenges in mobility, efficiency, and stability remain, ongoing advances in molecular design, device engineering, and hybrid integration are paving the way for the practical adoption of MPc-based photodetectors in flexible and emerging optoelectronic systems.

## 4. Gas Sensor

Beyond their photoelectric functionalities, MPcs possess a unique combination of chemical and physical properties, including high chemical stability, structural tunability, and extended conjugated π-electron systems. These attributes collectively facilitate a sensitive chemiresistive response at room temperature, rendering them highly attractive for gas sensing [81]. The underlying sensing mechanism primarily involves the adsorption of target gas molecules onto the MPc film, which modulates the charge-carrier density and consequently induces a measurable change in electrical conductivity. This response is often further enhanced by selective coordination between the gas molecules and the central metal ion of the MPc [82]. In contrast to conventional gas sensors, MPcs-based sensors offer several distinct advantages: efficient room-temperature operation (significantly reducing power consumption), enhanced selectivity through tailored molecular design, compatibility with flexible substrates, and low-cost fabrication [83]. To date, a variety of MPc-based sensors have been designed and developed for detecting different gases, such as NH_3_, NO_2_, and H_2_S.

### 4.1. NH_3_ Sensors

Owing to its typical reducing property, NH_3_ can withdraw electrons from the π-electron system of MPc molecules, thereby increasing the hole concentration in p-type MPc films and enhancing their electrical conductivity. Currently, most high-sensitivity NH_3_ sensors have been developed using thin-film architectures [84].

**Figure 7:**
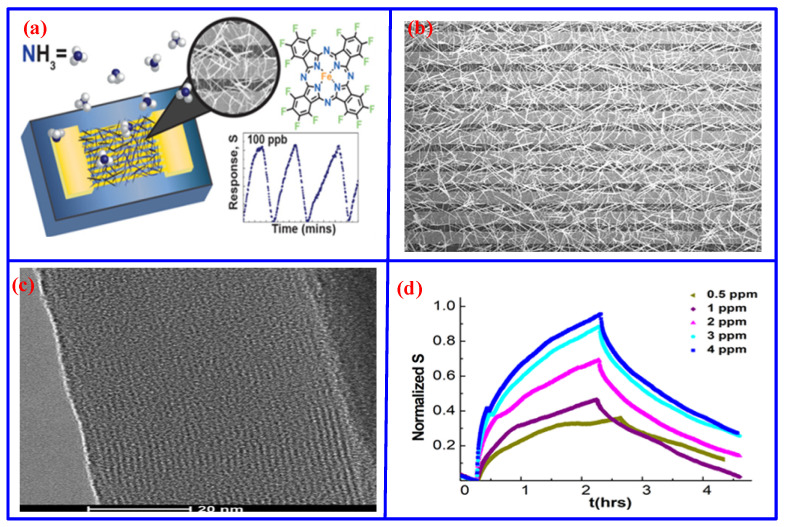
(**a**) Schematic diagram and recovery curve of F_16_FePc-based NH_3_ sensor [88]. (**b**) SEM image of F_16_FePc nanowires coverage of electron [88], (**c**) TEM image of single nanowire [88], and (**d**) response time of sensor at different NH_3_ concentrations [88].

However, such configurations often suffer from inherent limitations, including low specific surface area, lattice mismatch, and structural defects, all of which can degrade sensing performance. To address these issues, doping strategies have been explored to improve detection sensitivity [85]. Although effective, these approaches typically involve challenges in precisely controlling doping concentrations and high economic costs. To further improve sensitivity and reduce cost, NH_3_ sensors based on various MPc structures have been increasingly explored [86,87]. For instance, Flores et al. fabricated an NH_3_ sensor by directly growing F_16_FePc nanowires onto interdigitated electrodes via physical vapor transport (Figure 7a) [88]. As shown in Figure 7b,c, the F_16_FePc nanowires uniformly cover the electrode surface, providing a high surface area that not only enhances sensitivity and selectivity toward NH_3_ but also improves response and recovery speeds. The devices achieved a rapid response times (approximately 12 min at 100 ppb, 20 min at 40 ppb, and only a few seconds at 25 ppm), along with a low detection limit of 5 ppb, attributed to the efficient gas-nanowire interfacial interaction. Moreover, during three hours of exposure to varying NH_3_ concentrations, the responsivity of the sensor exhibited a marked dependence on concentration, indicating good sensitivity, as shown in Figure 7d. Similarly, Gai et al. developed an NH_3_ sensor by immobilizing TfmoPcCo onto polypyrrole-coated multi-walled carbon nanotubes (MCNT@PPy/TfmoPcCo), achieving fast response times (8 s to 10 ppm and 11.7 s to 50 ppm), a low detection limit of 11 ppb, and excellent stability and repeatability [89]. Further studies have examined the influence of gate pressure on sensor sensitivity. Gallardo-Pascual et al. evaluated the response of CuPc and F_16_CuPc films to NH_3_ under different gate pressures [90]. The LODs were determined to be 0.4 ppm for CuPc and 0.21 ppm for F_16_CuPc, highlighting that the sensing performance significantly depended on the gate pressure.

### 4.2. NO_2_ Sensors

In contrast to NH_3_, NO_2_ is a strong electron-accepting (oxidizing) gas. Upon adsorption onto an MPc film, NO_2_ extracts electrons from the π-electron system, thereby reducing the concentration of majority charge carriers (holes in a p-type semiconductor) and leading to a measurable increase in the electrical resistance of the film [91]. Moreover, the sensitivity and selectivity can be further enhanced through specific coordination interactions between NO_2_ and the central metal ion of the MPcs. Compared to conventional NO_2_ sensors, MPc-based NO_2_ sensors offer key advantages, including high sensitivity, low detection limits, excellent selectivity, room-temperature operation, and facile processability [92]. Owing to their favorable physicochemical properties, direct charge-transfer-based detection mechanism, and practical processing benefits, MPcs represent a highly promising class of materials for sensitive, selective, and low-power NO_2_ monitoring applications [93].

**Figure 8:**
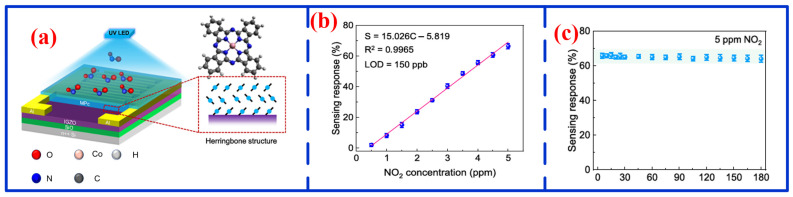
Schematic diagram of CoPc/IGZO sensor [97]: (**a**) architecture of device, (**b**) sensing response, and (**c**) environmental stability.

In recent years, various MPc-based sensors have been developed for NO_2_ detection [94,95]. For instance, Zhu et al. fabricated a dual-functional sensor based on vanadyl-phthalocyanine (VOPc) capable of simultaneously detecting NO_2_ and humidity [96]. With a 10 nm-thick VOPc layer, the sensor achieved a responsivity of 4710% under 10 ppm NO_2_, along with low baseline drift and short response/recovery times, demonstrating clear sensitivity and selectivity toward both NO_2_ and water vapor. The underlying mechanism involves opposing electrical behaviors: water molecules promote hole-electron recombination in VOPc, thereby reducing hole carrier density and increasing resistance, whereas NO_2_ adsorption extracts electrons, raising the hole concentration and decreasing resistance. Jaisutti et al. developed a CoPc/IGZO heterojunction NO_2_ sensor (Figure 8a) [97]. Under ultraviolet illumination, the device showed high sensitivity and rapid response to NO_2_. As shown in Figure 8b, the sensor current increases linearly with NO_2_ concentration over the range of 0–5 ppm, achieving the LOD of 150 ppb. In addition to these advantages, the sensor also exhibited outstanding environmental stability (Figure 8c). Furthermore, Isci et al. designed a sensor based on an electron-rich structure (TT-Pc) comprising thieno [3,2-b] thiophene (TT) and phthalocyanine (Pc) for the detection of both NO_2_ and SO_2_ [98]. At 20 ppm, the device showed response rates of 90% toward NO_2_ and 60% toward SO_2_, along with remarkable environmental stability, exhibiting less than 7% degradation after nine months of storage in air. Despite these notable advantages, MPc-based NO_2_ sensors face several practical limitations, including susceptibility to humidity interference, limited long-term stability under continuous operation, and often sluggish recovery at room temperature. Furthermore, their performance is highly dependent on film morphology and fabrication methods, which can affect device reproducibility. Therefore, achieving high selectivity in complex gas mixtures remains a significant challenge that must be addressed to facilitate practical deployment.

### 4.3. H_2_S Sensors

Hydrogen sulfide (H_2_S) is a highly toxic, colorless, dangerous, and ammable gas with a characteristic rotten-egg odor. Even at low concentrations, H_2_S can harm the human respiratory and nervous systems, while long-term or high-level exposure may lead to dizziness, vomiting, and even death [99]. Therefore, real-time monitoring and accurate detection of H_2_S have attracted substantial research interest. To date, various sensing technologies have been developed for H_2_S detection, including electrochemical [100], piezoelectric [101], metal oxide semiconductor [102], micro-electromechanical [103], and optical waveguide sensors [104]. However, their practical application is often limited by harsh operating conditions, weak gas adsorption, and poor sensitivity and selectivity [105].

**Figure 9:**
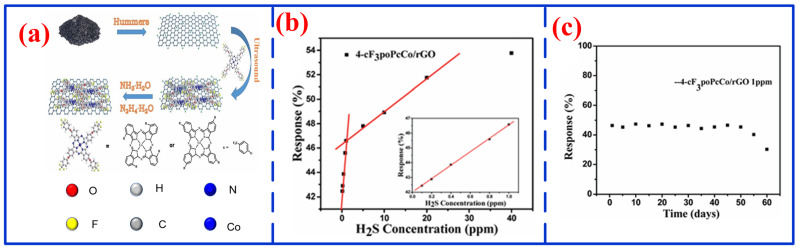
Schematic diagram of 3(4)-cF3po-PcCo/rGO hybrid structure [113]: (**a**) synthesis procedure; (**b**) response at different H_2_S concentrations within 0.1–1 ppm and 1–20 ppm; (**c**) response on different days.

In contrast to conventional sensing materials and configurations, MPcs, as a prominent class of metal–organic molecular materials, exhibit remarkable sensing performance. Recently, diverse MPc-based configurations have been designed and developed to achieve high stability and good selectivity for H_2_S detection [106]. For instance, Li et al. experimentally investigated the adsorption behavior of H_2_S on CuPc thin-film transistors, demonstrating that CuPc-based OTFT with a 195 nm insulator layer exhibits promising potential as H_2_S sensors [107]. To further improve detection limits and response times, Ivanova et al. fabricated H_2_S sensors by cross-linking single-walled carbon nanotubes (SWCNT) with ZnPc and CoPc, respectively [108]. The resulting SWCNT/CoPc composite showed a response time four times faster than that of SWCNT/ZnPc, with LOD of 18 and 65 ppb, respectively. The SWCNT framework provides more attachment sites for H_2_S molecules, significantly enhancing electron-exchange efficiency between H_2_S and MPcs, thereby shortening both response and recovery times. Building on these advances, Wang et al. constructed hybrid structures comprising tetra-α(β)-(4-trifluoromethylphenoxy)phthalocyanine cobalt and reduced graphene oxide (3(4)-cF3po- PcCo/rGO) via non-covalent modification for H_2_S detection, as illustrated in Figure 9a [109]. Utilizing strong π–π stacking interactions between the rGO surface and the extended π conjugated system of PcCo, the hybrid sensor achieved a sensitivity of 46.58 toward 1 ppm H_2_S and a room-temperature LOD of 11.6 ppb, along with excellent linearity over the range of 0.1–20 ppm and outstanding environmental stability, as shown in Figure 9b,c. Additionally, Wumier et al. developed a zinc phthalocyanine (ZnPc) film-based K^+^-exchanged optical waveguide (OWG) sensor for H_2_S detection [110]. The device exhibited excellent H_2_S-sensing performance at room temperature, characterized by a wide linear range (0.1 ppm–500 ppm), good reproducibility, high stability, and a low LOD.

Despite MPc-based gas sensors showing considerable promise due to their high sensitivity and tunable selectivity, they still face significant challenges, including limited long-term stability under real-world conditions, insufficient selectivity in complex gas mixtures, slow response/recovery times at room temperature, and susceptibility to environmental interference like humidity. To address these limitations, current research strategies focus on molecular engineering of the MPc core or peripheral substituents to enhance specific gas interactions, nanostructuring MPcs into thin films, nanowires, or quantum dots to increase active surface area, forming composites with conductive materials (e.g., graphene, carbon nanotubes, metal oxides) to improve charge transport and structural stability, and developing advanced sensor arrays integrated with pattern recognition algorithms for better discrimination among gases. Future research directions are geared toward creating intelligent, self-calibrating sensor systems compatible with the Internet of Things (IoT), utilizing machine learning for real-time and high-accuracy analysis of complex gas signatures, exploring biocompatible and flexible MPc configurations for wearable health and environmental monitors, and advancing scalable, low-cost fabrication techniques such as inkjet printing to enable widespread deployment in applications ranging from smart homes and industrial safety to personalized medical diagnostics.

## 5. Biosensors

Similar to the gas-sensing principle, biosensors serve as analytical devices that integrate a biological recognition element with a physicochemical transducer. Known for their high specificity, sensitivity, and rapid response, these sensors play a crucial role in fields such as disease diagnosis, environmental monitoring, food safety, and biodefense [111]. Conventional biosensors, however, primarily rely on the specific binding between a biological recognition element (e.g., an enzyme) and its target to generate a measurable signal. This approach faces inherent limitations, including the instability of biological components, slow response times, insufficient sensitivity, and challenges in miniaturization [112]. Owing to their unique electrochemical and structural properties, MPcs have emerged as promising candidate materials for diverse biodetection applications, offering a new pathway to overcome the constraints of traditional biosensor designs [113]. Currently, various MPc-based biosensors are being developed, particularly for cannabis and dopamine detection [114,115].

### 5.1. Cannabis Sensors

With the increasing global trend toward cannabis legalization, accurate monitoring of its primary active components, namely Δ^9^-tetrahydrocannabinol (THC) and cannabidiol (CBD), has become imperative for safeguarding public health and safety [116,117]. Conventional detection methods, typically based on chemical analysis and biochemical assays, often require sophisticated instrumentation, trained personnel, and time-consuming preparation, while suffering from a narrow detection window and the risks of false results. These limitations highlight the urgent demand for more robust, selective, and user-friendly sensing technologies. Owing to their superior molecular recognition and signal transduction capabilities, MPcs have emerged as highly effective materials for cannabis detection [118]. The central metal ions (e.g., Co, Fe) facilitate the redox reaction of the captured cannabis at significantly reduced overpotentials, thereby enhancing both sensitivity and selectivity by minimizing interference. This synergy between targeted molecular interaction and efficient electrocatalytic signal amplification positions MPcs as promising materials for developing sensitive, rapid, and reliable cannabis detection platforms [119].

**Figure 10:**
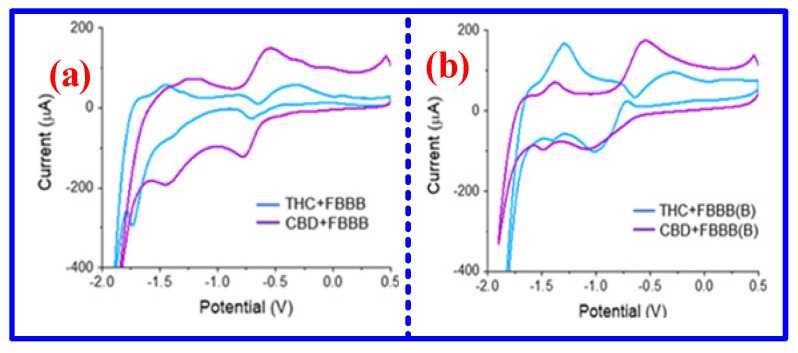
Current response of cobalt phthalocyanine to different mixed solutions [122]: (**a**) THC/CBD + FBBB; (**b**) THC/CBD + FBBB(B).

Currently, several MPc-based sensors have been developed to study the interaction between THC, CBD, and MPc films [120]. For example, organic thin-film transistors (OTFTs) incorporating MPcs have been designed for cannabis sensing, using molecular interactions between MPcs and THC or CBD [121,122]. The current magnitude changes in THC and CBD after the mixture of FBBB and FBBB(B) showed that CoPc exhibited pronounced sensitivity to the THC-FBBB complex, as shown in Figure 10. This behavior can be attributed to strong analyte-Pc coordination, accompanied by film restructuring to accommodate these interactions, demonstrating that both the physical and electrical characteristics of MPc films are closely intertwined with the nature of analyte coordination. These works indicate that analytes can rapidly induce physical changes in MPc films, which could have relevant implications for film engineering and other sensing applications. Additionally, Lamontagne et al. fabricated a series of novel R-AlPc-based OTFTs for detecting THC and CBD [123]. Among these, the 345F-AlPc-based sensor exhibited high sensitivity to THC vapor, attributed to pronounced changes in grain size, film roughness, thin-film reorganization, and crystal orientation. To further investigate the effects of deposition conditions, crystal morphology, and film thickness on THC-vapor sensitivity, Comeau et al. fabricated multiple MPc-based OTFTs under varied physical vapor deposition parameters [124]. By optimizing thin-film morphology, crystal polymorphs, and thickness, the sensitivity of the ZnPc-based device to THC vapor could be enhanced by up to 100-fold. Despite promising results, several challenges must be addressed to advance MPc-based cannabis sensors toward practical, real-world applications.

### 5.2. Electrochemical Sensors

Similar to cannabis sensors, electrochemical sensors operate by converting chemical interactions at an electrode surface into a quantifiable electrical signal. Their working principle is based on the redox reaction of a target analyte at the working electrode, which generates a measurable current or alters the interfacial potential or impedance [125]. However, conventional electrochemical sensors face several inherent limitations, such as selectivity challenges, electrode fouling and passivation, limited sensitivity and dynamic range, and operational complexities, hindering their practical deployment in portable or wearable sensing platforms [126,127]. Owing to their unique structural, electronic, and catalytic properties, MPcs have recently emerged as highly advantageous active materials for substance detection [128,129].

Currently, a wide range of MPc-based electrochemical sensors have been developed for detecting substances such as dopamine (DA), artemisinin, nifedipine, pesticides, and the antipsychotic drug promazine. For example, Natalia et al. constructed a composite sensor platform comprising NiTsPc, zinc oxide phthalocyanine particles, and carbon nanotubes immobilized on pyrolytic graphene for monitoring DA in human serum, which can achieve detection limits of 24 nM for chronoamperometry and 7.0 nM for differential pulse voltammetry (DPV) [130]. To further lower the detection limit, Peng et al. reported a highly sensitive DA sensor based on an indium tin oxide (ITO) electrode modified with ZnPc-P8BT-Pdots [131]. Under optimal conditions, this sensor detected DA over the range of 2.5 nM to 125 μM with an LOD of 1.69 nM (S/N = 3). Moreover, it was also successfully applied to determine DA concentration in hydrochloride injection, blood serum, and urine, with recoveries between 97.79% and 101.21%.

To broaden the application scope, Damphathik et al. developed a sensor using a glassy carbon electrode modified with a hybrid nanocomposite of cobalt phthalocyanine, graphene nanoplatelets (GNTs), multi-walled carbon nanotubes, and ionic liquids (ILs) for artemisinin detection [132]. This sensor exhibited a wide linear range (1.5–60 μM and 60–600 μM) with an LOD of 0.70 μM, along with good repeatability (2.9–3.0% RSD) and reproducibility (3.1–4.4% RSD), and satisfactory recoveries (105–116%) in drug and plant samples. Liu et al. designed an AlPc-CC POP-based electrochemical sensor for nifedipine (NIF) detection, which showed a linear response from 0.04 to 50 μM, an LOD of 0.0273 μM, and good reproducibility, stability, and selectivity [133]. Akyüz et al. constructed a sensor based on a hybrid of manganese phthalocyanine and polyaniline for pesticide detection, achieving low LODs (0.049, 0.088, and 0.062 mol dm^−3^ for fenitrothion, eserine, and diazinon, respectively), wide linear ranges, and high selectivity [134]. Additionally, Mounesh et al. developed a sensor for the antipsychotic drug promazine using a glassy carbon electrode (GCE) modified with ZnTEPZCAPC@MWCNT nanocomposites, achieving a broad linear range (0.05–635 μM), an ultralow LOD of 0.0125 nM, and excellent sensitivity, repeatability, and reproducibility [135]. Other high-performance MPc-based electrochemical sensors have also been reported, including mesoporous silica/titania with cobalt(II) phthalocyanine for pentachlorophenol detection [136].

**Figure 11:**
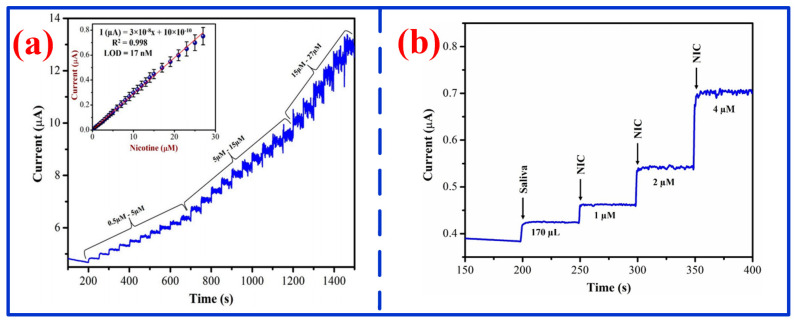
Current response of FePc/graphene for different nicotine concentrations and addition [143]: (**a**) nicotine concentration range of 0.5–2.7µM and (**b**) added different concentrations of nicotine.

**Figure 12:**
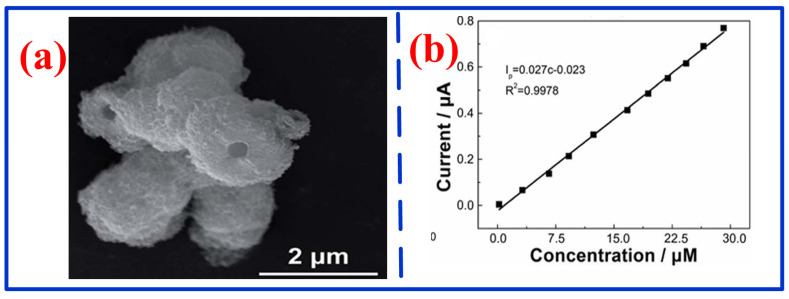
(**a**) SEM image of FePc MOF [144]; (**b**) current density at different vanillin concentrations [144].

To enable health assessment and scurvy prevention, recently, various MPc-based electrochemical sensors have been developed [137]. For instance, Sun et al. fabricated a sensor by depositing a solvent-processing CoPc (OC_8_H_9_)_8_ film on a glassy carbon electrode for AA detection [138]. Benefiting from its lamellar structure and large surface area, the sensor demonstrated excellent sensitivity and strong anti-interference capability, achieving a wide detection range of 0.1–100 μM and a low LOD of 36 nM. To further improve sensitivity and lower LOD, researchers have integrated metal phthalocyanine with MWCNTs, porous structures, or other carbon nanotubes to construct high-performance electrochemical sensors for AA or herbicide detection [139,140,141,142]. Additionally, Kamalasekaran et al. constructed an electrochemical nicotine sensor based on a glassy carbon electrode modified with iron phthalocyanine and graphene [143]. As shown in Figure 11a, the response current of this sensor varied linearly with time across this concentration range, indicating good linearity over the nicotine concentration range of 0.5–2.7µM with an LOD of 17 nM. Moreover, the response current gradually increases with nicotine concentrations, confirming strong concentration-dependent sensitivity (Figure 11b). Peng et al. fabricated an electrochemical vanillin sensor using an FePc MOF structure on a glassy carbon electrode (Figure 12a) [144]. As shown in Figure 12b, this sensor showed a wide, excellent linear response over the 0.22–29.14 μM range, with a low LOD of 0.05 μM, proving applicable for the practical detection of vanillin in human serum. To further enable self-powered operation, Zhang et al. developed an electrochemical sensor for hydrogen peroxide (H_2_O_2_) detection using graphene nanoplatelets (GNP)-modified FePc as the cathode catalyst and Ni as the anode material [145]. By mitigating the inherent aggregation and poor conductivity of iron phthalocyanine through interface engineering, the self-powered sensor attained a low LOD of 0.6 µM and a high sensitivity of 0.198 A/(M·cm^2^) for H_2_O_2_ detection.

To facilitate a comparative evaluation of sensing performance across different electrochemical sensors, Table 2 summarizes the limits of detection for various analytes achieved with different sensing materials. Compared with many inorganic materials, MPc-based electrochemical sensors consistently exhibit lower detection limits, which can be attributed to the efficient electron transfer enabled by the π-conjugated structure of MPcs. While carbon-based materials also support effective electron transport, their achievable sensitivity can be partially constrained by higher interfacial or charge-transfer barriers [139,140]. Moreover, compared to carbon materials, MPcs offer more well-defined and specific active sites, enabling high selectivity [128]. Despite their considerable promise, several challenges still hinder the widespread commercialization of MPc-based electrochemical sensors. To overcome these hurdles, current strategies focus on nanomaterial integration and interface engineering. Hybridizing MPcs with conductive nanostructures, such as graphene, carbon nanotubes, or metal nanoparticles, enhances electron transfer, increases active surface area, and improves stability. Molecular engineering of the MPc macrocycle or its peripheral substituents fine-tunes redox potential and interaction specificity toward target analytes. Advanced electrode architectures, including layer-by-layer assembly and controlled nanostructuring, help prevent fouling and ensure reproducible sensor performance. Additionally, coupling MPc-modified electrodes with selective membranes or pre-treatment steps can further improve selectivity in real-world samples.

Future research directions aim to advance MPc-based electrochemical sensors toward intelligent, integrated, and field-deployable analytical systems. Key trends include the development of flexible and wearable sensor platforms for continuous, non-invasive health monitoring (e.g., sweat or interstitial fluid analysis). Significant effort will also be directed toward scalable and precise fabrication techniques to produce low-cost, disposable sensor arrays with high reproducibility. The integration of machine learning for real-time multivariate signal analysis will enhance selectivity and enable pattern recognition in complex biological or environmental matrices. Furthermore, research will explore self-powered or energy-autonomous sensors, potentially coupled with microfluidic sampling, suitable for deployment in remote or resource-limited settings. Ultimately, the convergence of materials design, scalable manufacturing, and data-driven analytics is expected to propel the application of MPc electrochemical sensors into areas such as personalized medicine, environmental surveillance, and smart industrial monitoring.

## 6. Other Sensors

**Figure 13:**
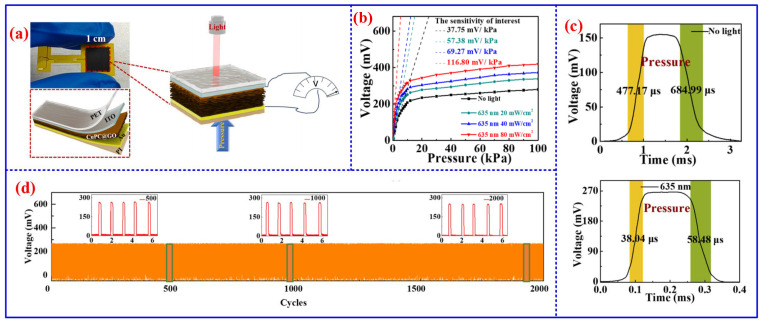
Structure and performance of CuPC@GO-based piezoelectric sensor [149]: (**a**) schematic diagram of structure, (**b**) sensitivity, (**c**) response time, and (**d**) long-term cyclic stability.

In addition to the previously discussed MPc-based sensors, other types, such as piezoelectric and humidity sensors, have also been developed. Generally, piezoelectric sensors convert mechanical stimuli (e.g., pressure, strain, or vibration) into measurable electrical signals. However, conventional piezoelectric sensors based on inorganic materials like lead zirconate titanate (PZT) often exhibit drawbacks, including rigidity, brittleness, and the presence of toxic elements. In contrast, the piezoelectric response in MPcs originates primarily from non-centrosymmetric molecular packing in specific crystal phases, along with a polar axis formed by the central metal ion and the surrounding macrocyclic ligand. Consequently, MPcs are regarded as promising candidates for next-generation piezoelectric sensors. For example, Wang et al. reported a light-enhanced, highly sensitive piezoelectric sensor based on a composite membrane of CuPc and graphene oxide (CuPC@GO) (Figure 13a) [149]. Under 635 nm illumination, the sensor delivered a higher output voltage of 381.17 mV at 50 kPa and an increased sensitivity of 116.80 mV/kPa in the low-pressure range (<5 kPa), achieving approximately two-fold and three-fold improvements over its performance in the dark (Figure 13b). Moreover, light illumination shortened the response time to 38.04 µs, enabling an ultra-fast response (Figure 13c), while maintaining 95% performance stability over 2000 loading-unloading cycles (Figure 13d). This improvement is attributed to photo-induced electron emission from CuPc molecules, which promotes greater electron transfer from CuPc to graphene, thereby enhancing polarization and pressure-sensing performance. Apart from piezoelectric sensors, Ngokpho et al. also developed a humidity sensor using chlorinated CuPc integrated with polyvinylpyrrolidone on flexible interdigitated electrodes [150]. The device achieved high sensitivity (1.2 × 10^4^%), excellent reproducibility (relative standard deviation < 5%), and a broad dynamic range (11–94%RH), making it suitable for wearable and unconventional sensing applications.

## 7. Summary and Prospect

In summary, MPcs represent a highly promising class of materials for advanced sensing applications, owing to their exceptional photoelectric properties and tunable molecular structures. This review systematically outlines recent progress in the development of MPc-based sensors, covering photodetectors, gas sensors, biosensors, and other sensors. The discussion begins with MPc-based photodetectors operating across the UV-Vis to NIR spectrum, with special attention given to heterojunction devices formed between MPcs and layered or nanowire-structured semiconductors. Then, advances in MPc-based gas sensors are examined, highlighting detection limits and response times achieved through diverse device architectures. Specifically, sensors utilizing nanowire or nanotube configurations show strong potential for highly sensitive detection of toxic and hazardous gases. Next, recent developments in MPc-based biosensors are summarized, focusing on the detection of analytes such as cannabis, dopamine, and ascorbic acid. For these sensors, doping and composite strategies have proven effective in improving sensitivity and lowering detection limits of these platforms. Finally, other types of MPc-based sensors have also been developed for piezoelectric and humidity sensing.

Despite notable progress, MPc-based sensors still face several key challenges that hinder their practical deployment, including limited long-term stability, insufficient selectivity, slow response and recovery kinetics, and susceptibility to environmental interference. To address these issues, current strategies focus on both the material and device-level innovations. Molecular engineering of the MPc core or its substituents is employed to enhance specific target interactions and electronic properties. Nanostructuring MPcs into thin films, nanowires, or quantum dots significantly increases the active surface area for analyte interaction. Forming composites or heterojunctions with conductive materials like graphene, carbon nanotubes, or metal oxides improves charge transport and stability, often introducing synergistic sensing capabilities. Additionally, the development of sensor arrays (electronic noses) combined with advanced pattern recognition algorithms helps overcome selectivity limitations by analyzing complex response signatures.

Future research directions are poised to transition MPc sensors from laboratory prototypes to integrated, intelligent systems. A key priority is to focus on the development of adaptive, self-calibrating sensor nodes that can be seamlessly incorporated into the Internet of Things for real-time environmental and industrial monitoring. The applications of machine learning and artificial intelligence will be crucial for real-time, high-fidelity analysis of complex data streams, enabling actionable insights. Another important direction involves developing biocompatible, flexible, and even biodegradable MPc-based formats for next-generation wearable health monitors and personalized medical diagnostics. Concurrently, advancing scalable, low-cost fabrication techniques, such as inkjet and roll-to-roll printing, is essential to enable their widespread deployment across applications ranging from smart homes and industrial safety to food quality assessment and public security.

## Figures and Tables

**Table 1:** Performance comparison of UV sensors based on different materials.

Material	Responsivity (A/W)	Detectivity (Jones)	Ref.
CoPc/Ga_2_O_3_	0.0184	1.92 × 10^17^	[65]
ZnO/CuPc	0.22711	7.63 × 10^11^	[66]
ZnO/GR	9.87 × 10^3^	-	[73]
CoPc/GaN	0.588	4.8 × 10^12^	[68]
CuPc/Cs_3_Bi_2_I_9_	1.96	6.91 × 10^11^	[72]
Ga_2_O_3_	0.05531	-	[74]
Au/SiC	2.025	6.53 × 10^11^	[75]

**Table 2:** The minimum detection limits for different substances in different materials.

Materials	Selectivity	LOD (μM)	Ref.
NiTsPc/ZnOPc/CNTs	DA	24 × 10^−3^ (Chronoamperometry) and 7.0 × 10^−3^ (DPV)	[130]
ZnPc-P8BT-Pdots	DA	1.69 × 10^−3^	[131]
CoPc/CNTs/GNTs	DA	0.70	[132]
AlPc-CC POP	NIF	0.0273	[133]
MnPc/Polyaniline	Pesticides	0.049 × 10^6^ (Fenitrothion), 0.088 × 10^6^ (Eserine), and 0.062 × 10^6^ (Diazinon)	[134]
ZnTEPZCAPC@MWCNT	Promazine	0.0125 × 10^−3^	[135]
CoPc(OC_8_H_9_)_8_	AA	36 × 10^−3^	[138]
FePc	H_2_O_2_	0.6	[145]
NC@ZIF-8	Folate	0.011	[146]
PbFe_12_O_19-NPs_/GCE	AMX	1.64 × 10^−3^	[147]
AuNPs/PHCQE/GPE	Ioniazid	0.31	[148]

## Data Availability

The data presented in this study are available in the manuscript.
